# Selection and Validation of Appropriate Reference Genes for qRT‐PCR Analysis of *Iris germanica* L. Under Various Abiotic Stresses

**DOI:** 10.1002/fsn3.4765

**Published:** 2025-01-12

**Authors:** Yuan Yuan, Chungui Liu, Jianzhong Bao, Fengtong Li

**Affiliations:** ^1^ Jiangsu Lixiahe District Institute of Agricultural Sciences Yangzhou Jiangsu China

**Keywords:** abiotic stress, *Iris germanica* L., quantitative real‐time PCR, reference gene

## Abstract

Choosing the appropriate reference genes for quantitative real‐time PCR (qRT‐PCR) is very important for accurately evaluating expression of target genes. *Iris germanica* L. is a widely used horticultural plant with high ornamental value, which also shows a strong ability to tolerate abiotic stresses. No comprehensive research has been carried out on optimal reference genes in *Iris germanica* L. under abiotic stress. In this study, nine reference genes were selected as candidates based on the transcriptome sequencing data of *Iris germanica* L. The assessment of expression stability under various abiotic stress was conducted using four distinct methods (GeNorm, NormFinder, BestKeeper, and RefFinder). It was found that the optimal reference genes were *ACT* and *F3H* for drought and different temperature stresses. *EF1α* and *ACT* exhibited superior performance under salt stress. The expression of the *IgP5CS* gene was evaluated to provide additional validation for the accuracy of the selected optimal reference genes, indicating that inappropriate may lead to significant deviations in the results. This research identified reliable reference genes in *I. germanica* L. across various abiotic stress conditions, thereby facilitating the investigation into the molecular mechanisms responsible for stress tolerance in *I. germanica* L.

## Graphical abstract

In this research, we evaluated the expression stability of 9 candidate reference genes by four different algorithms (GeNorm, Normfinder, BestKeeper, and RefFinder). *ACT* and *F3H* appeared to be the most reliable reference genes under different abiotic stresses. Furthermore, the expression patterns of *IgP5CS*‐validated *ACT* and *F3H* genes can serve as appropriate reference genes.

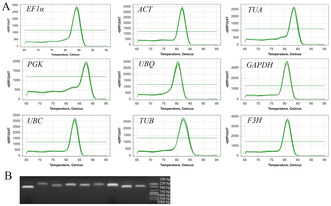

## 1 Introduction

Quantitative real‐time PCR (qRT‐PCR) is a widely employed technique for revealing gene function, due to its high sensitivity and precision (Bustin 2002; Gachon, Mingam, and Charrier 2004). Nevertheless, the accuracy of gene expression can be significantly influenced by various factors including the sample quality, and the amplification efficiency of qRT‐PCR (Derveaux, Vandesompele, and Hellemans 2010; Hao et al. 2014). To mitigate the impact of these confounding factors, internal reference genes are commonly employed in experimental settings to ensure precise and biologically relevant expression measurements (Schmidt and Delaney 2010; Zhao et al. 2022). Housekeeping genes are frequently employed as standardized reference genes (Jain et al. 2006; Souček et al. 2017), nevertheless, their expression levels exhibit significant variations across diverse tissues and species (Wang et al. 2021). Furthermore, several reports have demonstrated that environmental factors such as salinity, temperature, and hormones, along with particular experimental conditions, can also affect their expression levels (Podevin et al. 2012; Xu et al. 2015; Silva et al. 2021). Unstable reference genes may lead to substantial distortions and misrepresentations of the transcript data (Remans et al. 2014). To ensure the accuracy of expression data, it is crucial to choose appropriate reference genes according to different plant species and various experimental conditions.

*Iris germanica* L., which is a widely used perennial flower, has high ornamental value with big and colorful flowers. It was also applied in the cosmetics, pharmaceutics, and perfumes industries (Khatib, Faraloni, and Bouissane 2022). Furthermore, the rhizome extract from *I. germanica* L. exhibited a strong effect on pesticidal and anti‐cancer activities (Iranzadasl et al. 2021). *I. germanica* L. also showed a higher tolerance to drought and cold compared with the other species of the genus *Iris* (Zhang et al. 2021). The regulatory mechanism responsible for the adaptation to abiotic stresses in *I. germanica* L. is currently unknown. The impact of abiotic stresses on gene expression levels can be complicated and diverse (Zhang et al. 2021). To date, although a report has demonstrated that *IgUBC*, *IgGAPDH*, and *IgTUB* were appropriate reference genes for *I. germanica* L. across different flowering stages (Wang et al. 2021), the optimal reference genes for various abiotic stresses have not been elucidated.

This research aimed to discover highly stable reference genes in *I. germanica* L. under various abiotic stress conditions. 9 candidate reference genes, *TUA*, *TUB, ACT*, *EF1α*, *PGK*, *UBQ*, *GAPDH*, *UBC*, and *F3H*, generated from the transcriptome database were investigated using qRT‐PCR under drought, salt, heat, and cold treatments. Four statistical algorithms (GeNorm, NormFinder, BestKeeper, and RefFinder) have been applied to evaluate the optimal reference genes needed for normalization. To confirm the reliability and validity of the reference genes screened, the expression of *IgP5CS* (△1‐Pyrroline‐5‐Carboxylate Synthetase) gene encoding the crucial regulatory enzymes in the proline biosynthesis pathway which participates in stress tolerance (Yang et al. 2021) was analyzed under the above conditions. Our research will contribute to gene expression and help accelerate the comprehension of abiotic stress mechanisms in *I. germanica* L.

## 2 Materials and Methods

### 2.1 Plant Materials and Treatments

**FIGURE 1:**
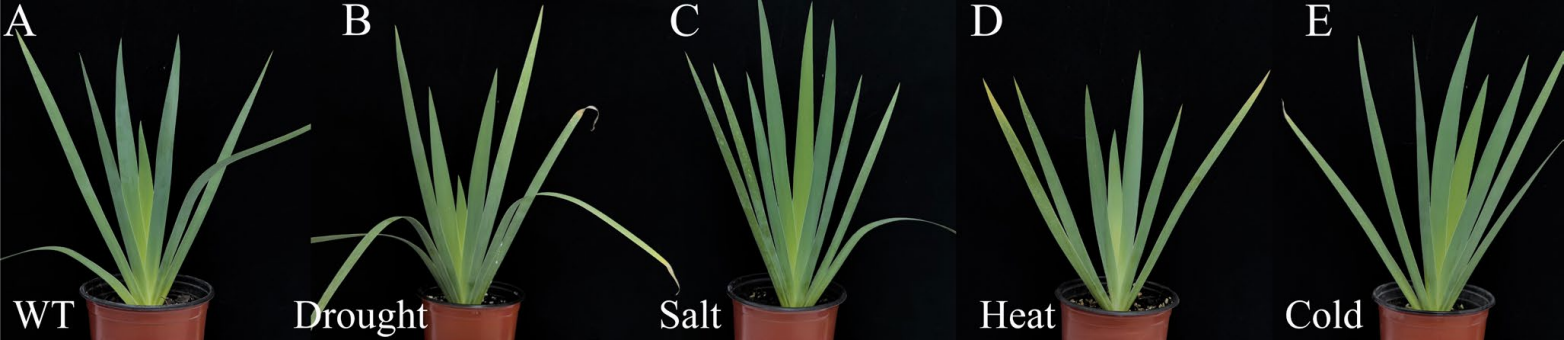
The phenotype of the abiotic stress treated plants. The phenotype of the plants treated with different abiotic stresses for 72 h. (A) WT, (B) drought stress, (C) salt stress, (D) heat stress, and (E) cold stress.

The plant materials used for the experiments were *I. germanica* cultivar ‘October sunshine’, which were collected from the Iris Resource Nursery (32°25'N; 119°23'E) at Jiangsu Lixiahe District Institute of Agricultural Sciences, China. Seedlings (Figure 1) were selected from tissue‐cultural plantlets at the age of 6 months and planted in an artificial climate incubator under normal conditions (25°C, 16 h light/8 h dark). For cold and heat treatments, plants were moved into the artificial climate incubator at 6°C and 40°C, respectively. For drought and salt stress treatments, the roots of plants were watered with full‐strength Murashige and Skoog medium solution supplemented with 250 mM NaCl or 20% PEG 6000, respectively. Leaves were then collected at 0, 4, 12, 24, 48, and 72 h after various stress treatments, and then placed in liquid nitrogen immediately. All samples were stored at −80°C for subsequent experiments. Each experiment included three biological replicates.

### 2.2 Reference Gene Selection

The sequences of reference genes reported from *Arabidopsis thaliana* and *Oryza sativa* were obtained from The Arabidopsis Information Resource (TAIR) and Rice Genome Annotation Project (RGAP). These sequences were used to perform a blast search their homologues from the transcriptome assemblies in *I. germanica* L. 9 candidate reference genes (*EF1α*, *TUA*, *TUB*, *UBQ*, *UBC*, *GAPDH*, *PGK*, *ACT*, *F3H*) and one target gene (*P5CS*) were obtained from the *I. germanica* L. transcriptome data from our laboratory (unpublished). Their identity to known amino acid sequences were ranging from 54.91% to 98.94% (Table 1). Primer designs were conducted using Primer 5.0 software. Parameters were set as follows: melting temperatures (Tm) 55°C–65°C, primer lengths 17–25 bp, and PCR product lengths 107–295 bp (Table 2). All PCR products were verified by 1.5% agarose gel electrophoresis.

**TABLE 1:** Similarity to genes from *Arabidopsis thaliana* and *Oryza sativa* .

Gene	*Arabidopsis thaliana* locus	Amino acid identity with *I. germanica* (%)	*Oryza sativa* locus	Amino acid identity with *I. germanica* (%)
*IgEF1α*	At1G07940	95.77	Os03g08050	97.09
*IgGAPDH*	AT3G04120	64.40	Os02g07490	85.55
*IgACT*	At5g09810	98.94	Os01g64630	96.83
*IgUBQ*	At4G05320	84.92	Os02g06640	85.10
*IgUBC*	At5G53300	97.97	Os06g30970	95.95
*IgPGK*	At1G56190	90.20	Os05g41640	87.40
*IgTUA*	At4G14960	97.35	Os03g51600	97.35
*IgTUB*	At1g75780	97.54	Os06g46000	96.89
*IgF3H*	At3G51240	85.33	Os03g03034	54.91
*IgP5CS*	AT2G39800	86.45	Os05g38150	87.95

**TABLE 2:** Primer sequences and amplification parameters.

Gene	Description	Primer sequences (forward/reverse)	Product length (bp)	*R* ^2^	Amplification efficiency (%)
*IgEF1α*	Elongation factor 1 alpha	CCCCTCCGTCTTCCTCTTC	284	0.999	97.96
		TTGGCAGCCTCCTTTGC			
*IgGAPDH*	Glyceraldehyde3‐phosphate dehydrogenase	CCCGTCTTGCCTGTCATTAG	152	0.991	103.54
		ATCTGAACTGTTCCCGTCTCC			
*IgACT*	Actin	TGGTGGTTGAGAAGACTGGG	177	0.997	99.05
		TGGCACGGATTCGGGAG			
*IgUBQ*	Ubiquitin	CTCCGGCTTAGAGGTGGTATG	119	0.993	91.89
		TCTTTGTCCTGGATCTTTGCTT			
*IgUBC*	Ubiquitin‐protein ligase	GCGGCGTCTTCCTCGTTA	235	0.992	103.09
		TGAGCAATCTCAGGCACCAA			
*IgPGK*	Phosphoglycerate kinase	TGGACGACGCCCAGAAC	156	0.993	102.52
		GCACAAGAGGAGCCAAACTAA			
*IgTUA*	Alpha‐tubulin	GACCTACACTAACCTCAACCGTCT	139	0.991	101.26
		CATGAAGTGGATTCTCGGGTAC			
*IgTUB*	Beta‐tubulin	TGGATTCCCAACAATGTCAAGTC	201	0.994	96.33
		CCGCCTCTGTAAACTCCATCTCA			
*IgF3H*	Flavanone 3‐hydroxylase	AGCAGAGGCAGCGTGTC	149	0.999	108.34
		CAGGTCCTCCGCCACA			
*IgP5CS*	Pyrroline‐5‐carboxylate synthase	GATTCCAAGAGGCAGTAACAA	277		
		AGGTCCACCACAAAGAGCA			

### 2.3 RNA Extraction and cDNA Synthesis

The total RNA of leaf samples was extracted using RNA simple Total Kit (TakaRa Dalian, China) following the product manual. The extracted RNA was assessed for quality and purity by NanoDrop 2000 spectrophotometer (Thermo, Wilmington, USA). RNA samples with an A260/A280 ratio of 1.9–2.1 and A260/A230 ratio > 2.0 were used for subsequent cDNA synthesis. cDNA was synthesized using 5 μg of total RNA in a volume of 20 μL with the PrimeScript RT cDNA Synthesis Kit (TaKaRa, Dalian, China). The initial reaction containing: 5 μg total RNA, 1 μL oligo (dt) primers, 1 μL dNTPs, and RNase‐free water. The mixture was incubated at 65°C for 5 min. The reverse transcription reaction: 10 μL RNA Mix, 4 μL 5 × PrimeScript buffer, 20 U RNase inhibitor, 200 U RT enzyme, and RNase‐free water. The mixture was incubated at 42°C for 30 min, followed by 70°C for 15 min. The cDNA was then diluted to a final volume of 60 μL with nuclease‐free water.

### 2.4 Quantitative Real‐Time PCR

The qRT‐PCR assay was implemented and analyzed with CFX96 system (Bio‐Rad). Each reaction assay was prepared as follows: 50 ng cDNA, 0.5 μL of each primer, 10 μL 2 × TransStart Tip Green aPCR SuperMix, and RNase‐free water. The amplification program was used: an initial step of 95°C for 30 s, 94°C for 5 s, and 60°C for 30 s (×40). The melting curves were measured after cycle 40 by heating from 60°C to 95°C at a rate of 0.5°C s^−1^. Each reaction was conducted for three replicates. The cycle threshold (Ct) value was collected automatically. Tenfold dilution cDNA series (10, 100, 1000, 10,000) were used to calculate the standard curve values. The amplification efficiency (*E*) and correlation coefficient (*R* ^*2*^) values were calculated by the formula *E* = [10^−1/slope^−1] × 100%.

### 2.5 Statistical Data Analysis

Three commonly used algorithms GeNorm, NormFinder, BestKeeper, and a web‐based tool, RefFinder (http://www.ciidirsinaloa.com.mx/RefFinder‐master/?type=refer%20ence#tabs‐1) were used to evaluate the stability of candidate reference genes under different conditions (Curis et al. 2019; Xie, Wang, and Zhang 2023; W. Xu et al. 2020). The GeNorm measures the stability of reference gene according to the *M* value. GeNorm also calculated the *V*_*n*_/*V*_*n*+1_ (pairwise variation) to determine the optimal number of reference genes needed. Reference genes are ranged with NormFinder according to their stability value (SV). BestKeeper program measures the expression stability of genes by assessing the coefficients of variation (CV) and the standard deviations (SD). The lower values of CV and SD are considered that the gene is more suitable to be used as references. Finally, the results obtained from the above three algorithms were integrated by the web‐based analysis tool RefFinder.

### 2.6 Validation of Selected Reference Genes

We quantified the expression of *IgP5CS* in all the tested samples to confirm the stabilities of the reference genes selected. Normalization of the gene *IgP5CS* was conducted using the two most stable and one most unstable reference genes as determined by RefFinder. The 2^−∆∆Ct^ method was used to analyze the relative gene expression level. Three technical replicates were performed for each biological sample. The bar plot was generated by the platform ImageGP 2 (https://www.bic.ac.cn/BIC) (Chen et al. 2024).

## 3 Results

### 3.1 Assessment of Primer Specificity of Reference Genes

**FIGURE 2:**
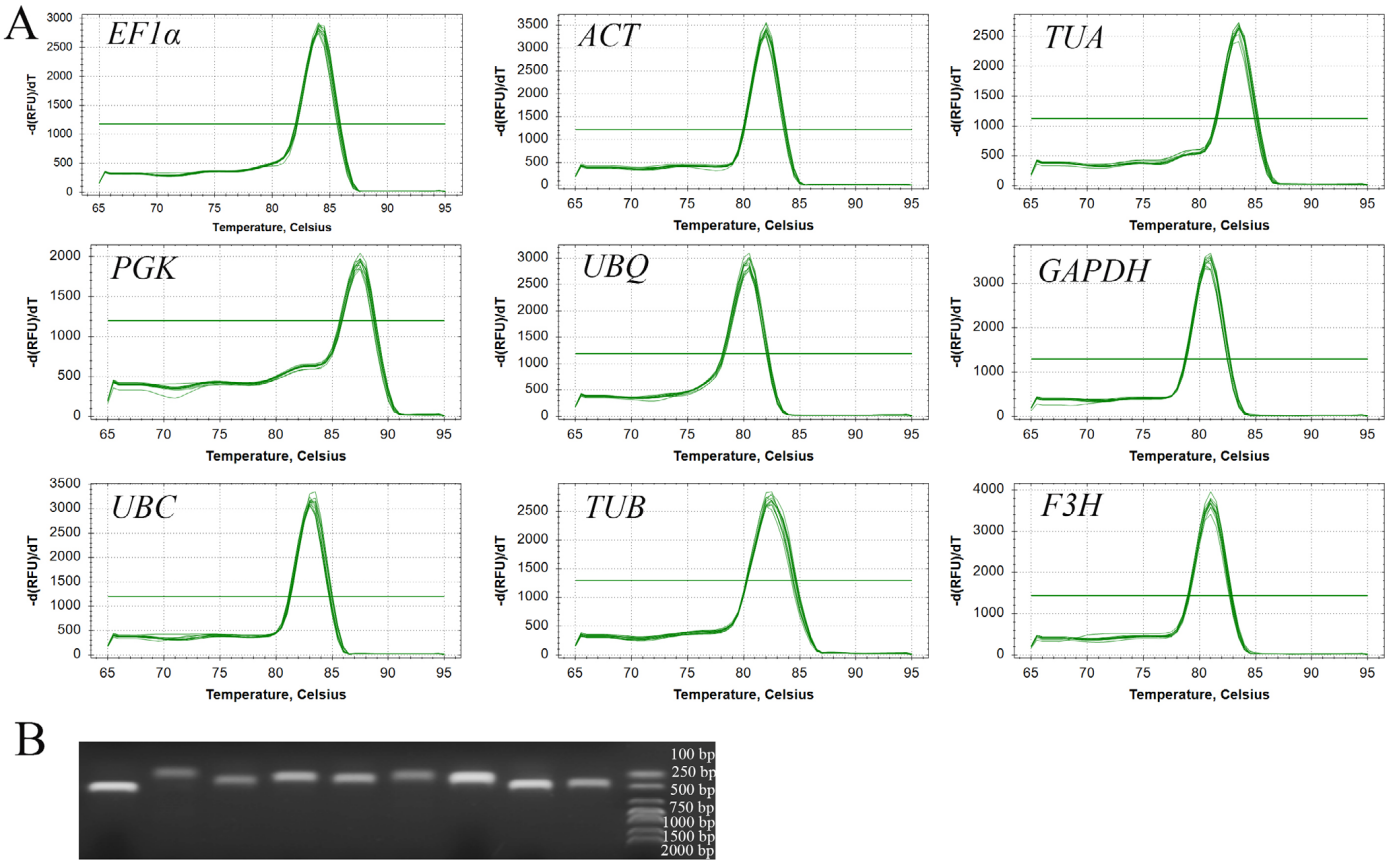
Primer specificity and amplicon size of 9 candidate reference genes. (A) melting curves of 9 candidate reference genes exhibiting single peaks. (B) agarose gel electrophoresis (1.5%) showing specific amplification products of expected size using qRT‐PCR. 1–9: *EF1α*, *UBQ*, *ACT*, *TUA*, *PGK*, *F3H*, *GAPDH*, *UBC*, and *TUA*. *M*: 2000 bp marker.

Only a specific product of the expected size was observed by 1.5% gel electrophoresis (Figure 2). The melt curves of all primers had a single amplification peak with no primer dimer appearing. The amplification efficiency of 9 reference genes ranged from 91.89% (*UBQ*) to 108.34% (*F3H*), which met the requirement of 90.00%–110.00%. The *R* ^2^ ranged from 0.991 to 0.999, which is consistent with *R* ^2^ > 0.980. These results suggest that the primers designed for the 9 reference genes have successfully met the standard for qRT‐PCR and are suitable for further experiments. The information regarding the candidate reference genes, as well as correlation coefficients, is shown in Table 2.

### 3.2 Expression Analysis of Reference Genes

**FIGURE 3:**
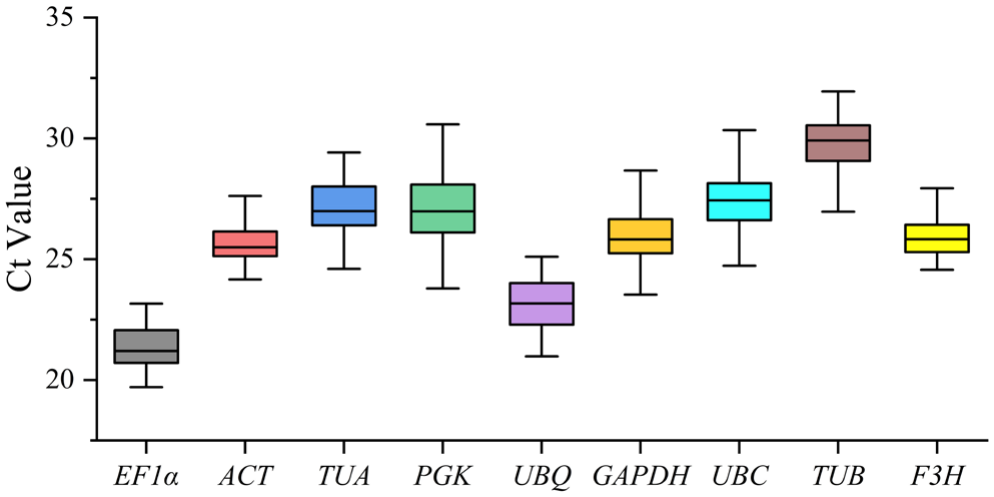
Expression levels of 9 candidate reference genes across all experimental samples. The boxes indicate the 25/75 percentiles and the lines across the box indicate the median values. The whisker caps indicate the maximum and minimum Ct values. The higher boxes and whiskers indicate the greater the variations.

Ct value is an important indicator of gene expression abundance, with genes exhibiting smaller Ct values demonstrating higher levels of expression. All Ct values ranged from 17.58 to 32.42 under different experimental treatments (Figure 3). The mean Ct ranged from 21.45 (*EF1α*) to 29.77 (*TUB*). The box plots illustrate the variation of Ct values, a decreased level of dispersion suggesting a higher degree of consistency in gene expression. *ACT* has the lowest variation trend, whereas *PGK* has the highest variation trend.

### 3.3 Expression Stability Analysis

#### 3.3.1 GeNorm Analysis

**FIGURE 4:**
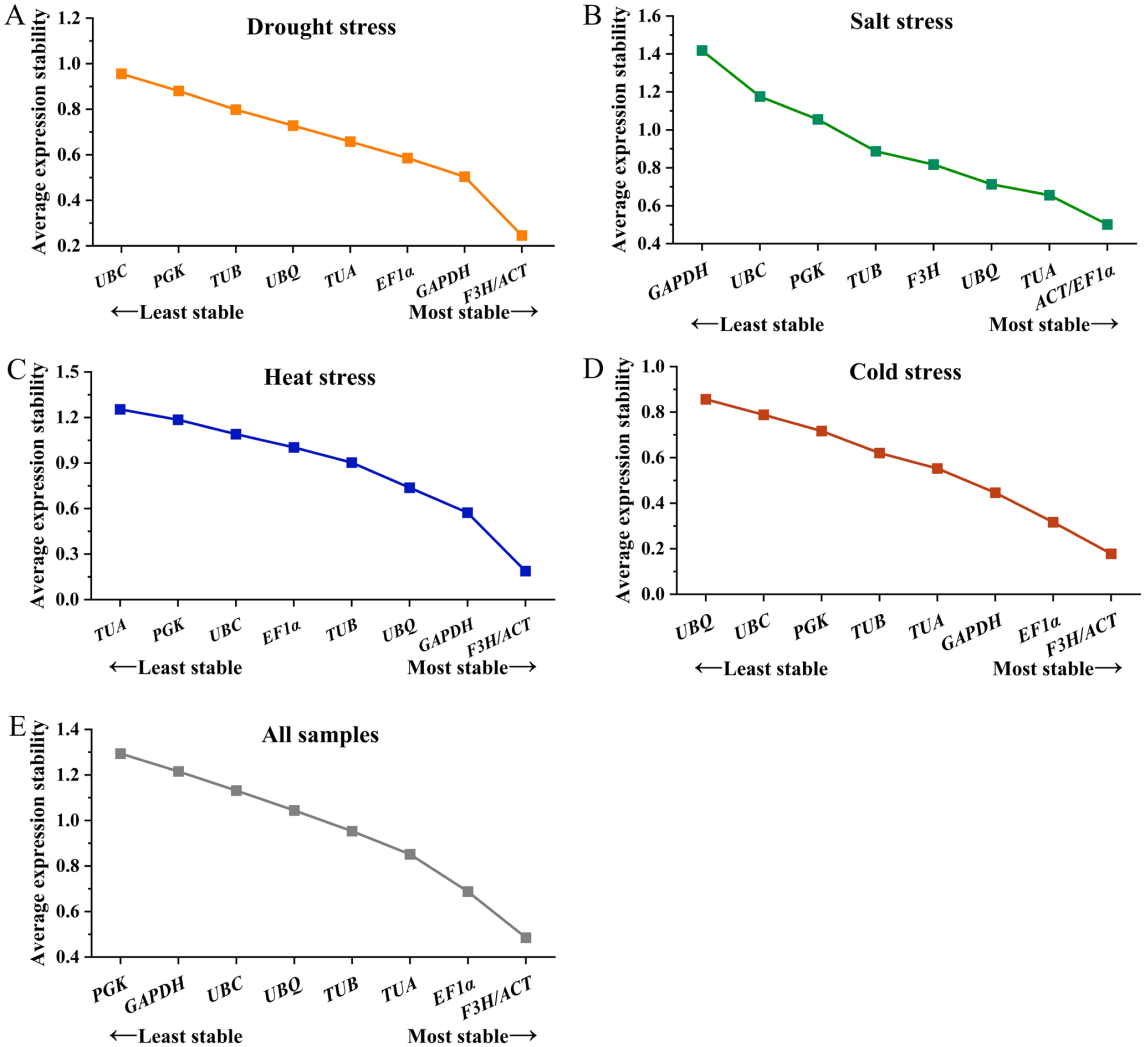
Expression stability of reference genes under different conditions based on GeNorm analysis. The most stable genes are on the right, whereas the least stable genes are on the left. (A) drought stress, (B) salt stress, (C) heat stress, (D) cold stress, and (E) all samples.

The default threshold of the *M* value was 1.5, whereas the smaller *M* value means greater stability. The *M* values of all reference genes were found to be less than 1.5, demonstrating that they met the fundamental criteria for serving as reference genes (Figure 4). For the drought treatment, the most stable reference genes were *ACT* and *F3H*, whereas the least stably expressed gene was *UBC*. *EF1α* and *ACT* (*M* = 0.50) showed the highest stability under salt stress, *ACT* and *F3H* (*M* = 0.19) showed the most stable expression levels under heat treatment. For the cold treatment, the expression levels were most stable for *ACT* and *F3H* (*M* = 0.18). For all samples, *F3H* and *ACT* (*M* = 0.49) were the most stably expressed genes.

**FIGURE 5:**
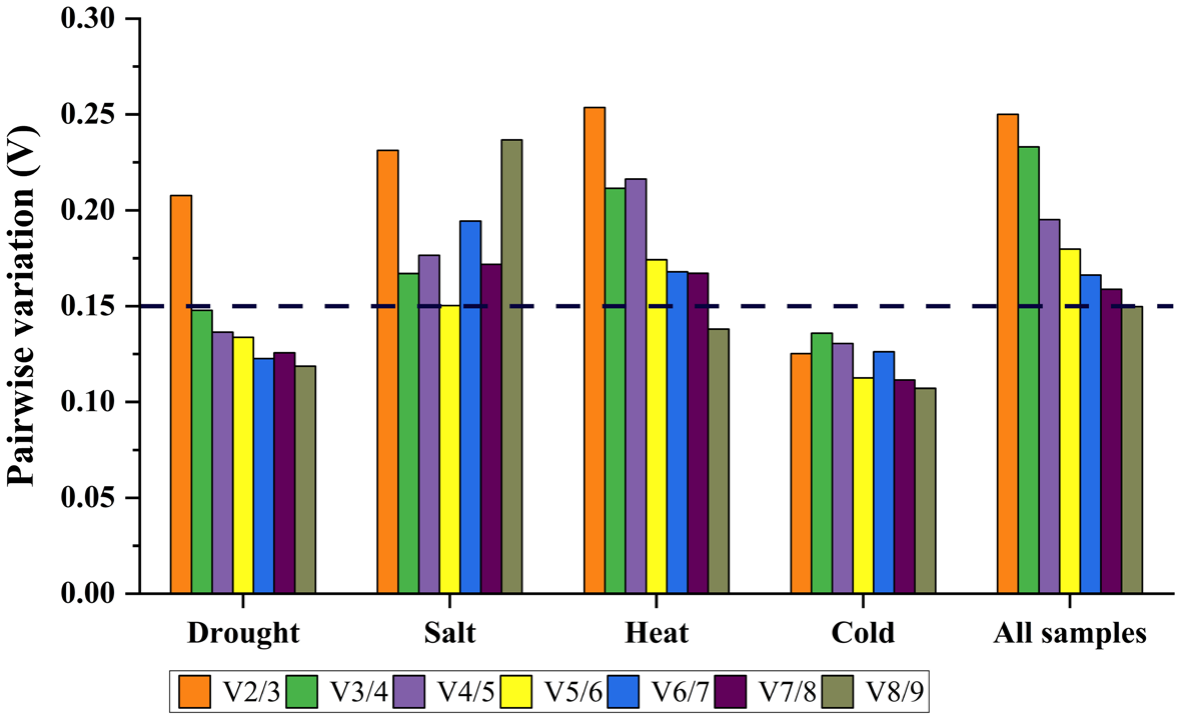
GeNorm analysis of the *V* values for the reference genes under various conditions. The average pairwise variations (*V*_*n*_/*V*_*n*_ + 1) were analyzed to measure the effect of adding reference gene on the qRT‐PCR. *V*_*n*_/*V*_*n*_ + 1: The pairwise variation between normalization factors *n* and *n* + 1.

GeNorm algorithm was employed to compute the *V*_*n*/*n*+1_ (pairwise variation), and the default *V*_*n*/*n*+1_ value is 0.15. For the drought stress samples, *V*_3/4_ was below 0.15 (Figure 5), suggesting that three reference genes are needed for normalizing gene expression. For the heat treatment, *V*_8/9_ was 0.14, showing that eight reference genes are required. For the cold treatment, *V*_2/3_ was 0.13, indicating that two reference genes were needed. For the salt treatment, all the *V*_*n*/*n*+1_ exceeded 0.15, that indicates the optimal number of reference genes has not been determined. For all the samples, *V*_8/9_ was 0.149, showing that eight reference genes are necessary.

#### 3.3.2 NormFinder Analysis

**FIGURE 6:**
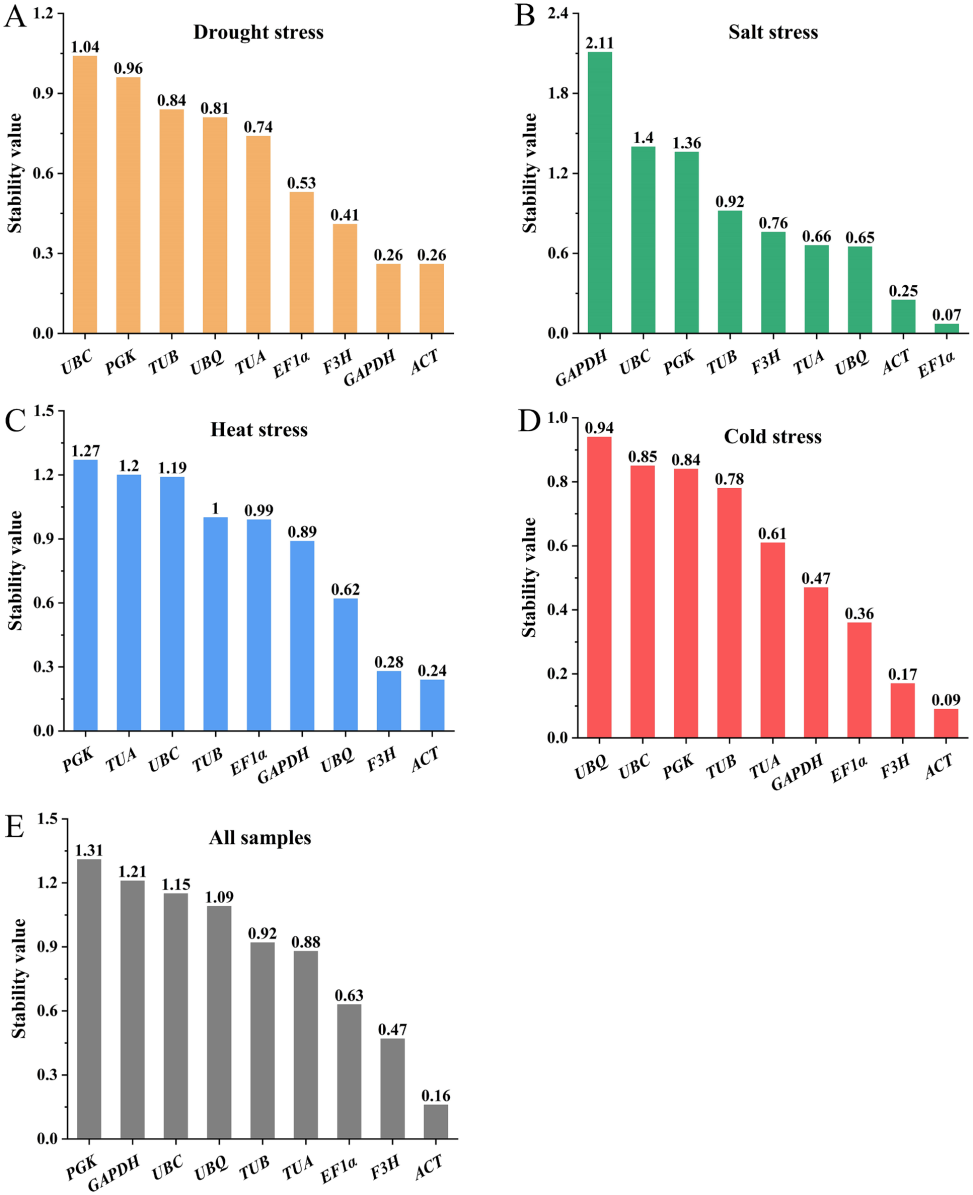
Ranging of reference genes using NormFinder software. (A) drought stress, (B) salt stress, (C) heat stress, (D) cold stress, and (E) all samples. The lower the stability value, the higher the expression stability.

NormFinder is employed to compute the stable value for each reference gene, as lower variation means greater gene expression stability. The results are displayed in Figure 6. For the drought treatment, *ACT* (0.26) and *GAPDH* (0.26) exhibited the highest stability. Regarding the salt treatment, *EF1α* (0.07) and *ACT* (0.25) exhibited the highest stability. The most stably expressed genes were *ACT* and *F3H* for the heat treatment, cold treatment and all samples, which was consistent with the results obtained from the GeNorm analysis.

#### 3.3.3 BestKeeper Analysis

**FIGURE 7:**
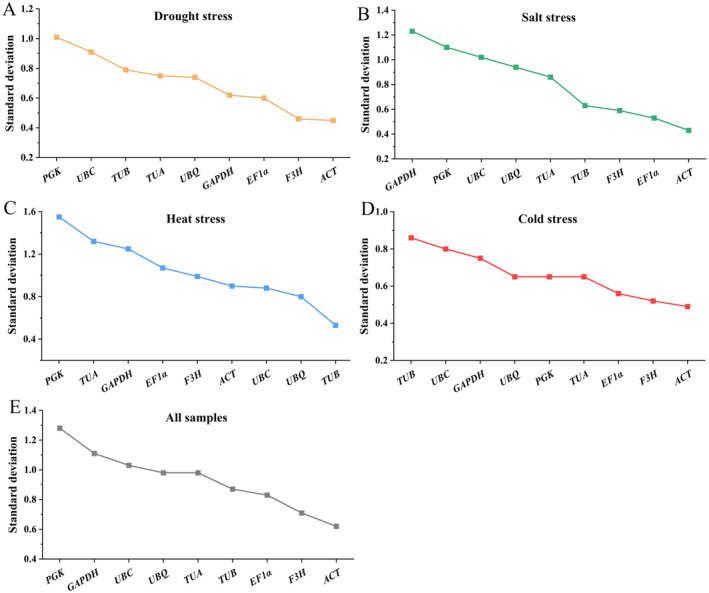
Stability evaluation of reference genes calculating by BestKeeper. (A) drought stress, (B) salt stress, (C) heat stress, (D) cold stress, and (E) all samples.

The standard deviation (SD) obtained from BestKeeper are showed in Figure 7. *ACT* and *F3H* had the most stable expression for the drought treatment, cold treatment and all samples. *ACT* and *EF1α* were the genes with the most stable expression under salt stress. For the heat treatment, *TUB* and *UBQ* were the most stable genes, which was discrepant with the results obtained from GeNorm and NormFinder analysis.

#### 3.3.4 RefFinder Analysis

To avoid limitations caused by a single program, ReFinder was employed to analyze the results of the above three algorithms. The rankings of RefFinder are shown in Table 3. The ranking was as follows: *ACT* > *F3H* > *EF1ɑ* > *TUA*>*TUB* > *UBQ*>*UBC* > *GAPDH*>*PGK*. *ACT* and *F3H* showed the highest stability for the drought treatment, heat treatment, cold treatment, and all the samples. For the salt treatment, *EF1ɑ* and *ACT* exhibited the highest stability, whereas *GAPDH* exhibited the least stability.

**TABLE 3:** Stability ranking of reference genes in RefFinder.

Method	1	2	3	4	5	6	7	8	9
Drought stress									
GeNorm	*ACT*|*F3H*		*GAPDH*	*EF1α*	*TUA*	*UBQ*	*TUB*	*PGK*	*UBC*
NormFinder	*ACT*	*GAPDH*	*F3H*	*EF1α*	*TUA*	*UBQ*	*TUB*	*PGK*	*UBC*
BestKeeper	*ACT*	*F3H*	*EF1α*	*GADPH*	*UBQ*	*TUA*	*TUB*	*UBC*	*PGK*
Comprehensive ranking	*ACT*	*F3H*	*GAPDH*	*EF1α*	*TUA*	*UBQ*	*TUB*	*PGK*	*UBC*
Salt stress									
GeNorm	*EF1α*|*ACT*		*TUA*	*UBQ*	*F3H*	*TUB*	*PGK*	*UBC*	*GAPDH*
NormFinder	*EF1α*	*ACT*	*UBQ*	*TUA*	*F3H*	*TUB*	*PGK*	*UBC*	*GAPDH*
BestKeeper	*ACT*	*EF1α*	*F3H*	*TUB*	*TUA*	*UBQ*	*UBC*	*PGK*	*GADPH*
Comprehensive ranking	*EF1α*	*ACT*	*TUA*	*UBQ*	*F3H*	*TUB*	*PGK*	*UBC*	*GAPDH*
Heat stress									
GeNorm	*ACT*|*F3H*		*GAPDH*	*UBQ*	*TUB*	*EF1α*	*UBC*	*PGK*	*TUA*
NormFinder	*ACT*	*F3H*	*UBQ*	*GAPDH*	*EF1α*	*TUB*	*UBC*	*TUA*	*PGK*
BestKeeper	*TUB*	*UBQ*	*UBC*	*ACT*	*F3H*	*EF1α*	*GADPH*	*TUA*	*PGK*
Comprehensive ranking	*ACT*	*F3H*	*UBQ*	*TUB*	*GAPDH*	*UBC*	*EF1α*	*TUA*	*PGK*
Cold stress									
GeNorm	*ACT*|*F3H*		*EF1α*	*GAPDH*	*TUA*	*TUB*	*PGK*	*UBC*	*UBQ*
NormFinder	*ACT*	*F3H*	*EF1α*	*GAPDH*	*TUA*	*TUB*	*PGK*	*UBC*	*UBQ*
BestKeeper	*ACT*	*F3H*	*EF1α*	*PGK*	*UBQ*	*TUA*	*GADPH*	*UBC*	*TUB*
Comprehensive ranking	*ACT*	*F3H*	*EF1α*	*GAPDH*	*TUA*	*PGK*	*TUB*	*UBQ*	*UBC*
All samples									
GeNorm	*ACT*|*F3H*		*EF1α*	*TUA*	*TUB*	*UBQ*	*UBC*	*GAPDH*	*PGK*
NormFinder	*ACT*	*F3H*	*EF1α*	*TUA*	*TUB*	*UBQ*	*UBC*	*GAPDH*	*PGK*
BestKeeper	*ACT*	*F3H*	*EF1α*	*TUB*	*UBQ*	*TUA*	*UBC*	*GADPH*	*PGK*
Comprehensive ranking	*ACT*	*F3H*	*EF1α*	*TUA*	*TUB*	*UBQ*	*UBC*	*GAPDH*	*PGK*

### 3.4 Reference Gene Validation

To evaluate the precision of the selected reference gene, we examined the expression of the *IgP5CS* gene under various stress conditions. *IgP5CS* is a stress‐inducible gene and plays a crucial role in enhancing plants abiotic stress tolerance. The two most stable reference genes (*ACT* and *F3H*) and the most unstable reference gene (*PGK*) were selected for normalizing gene expression data according to the RefFinder rankings.

**FIGURE 8:**
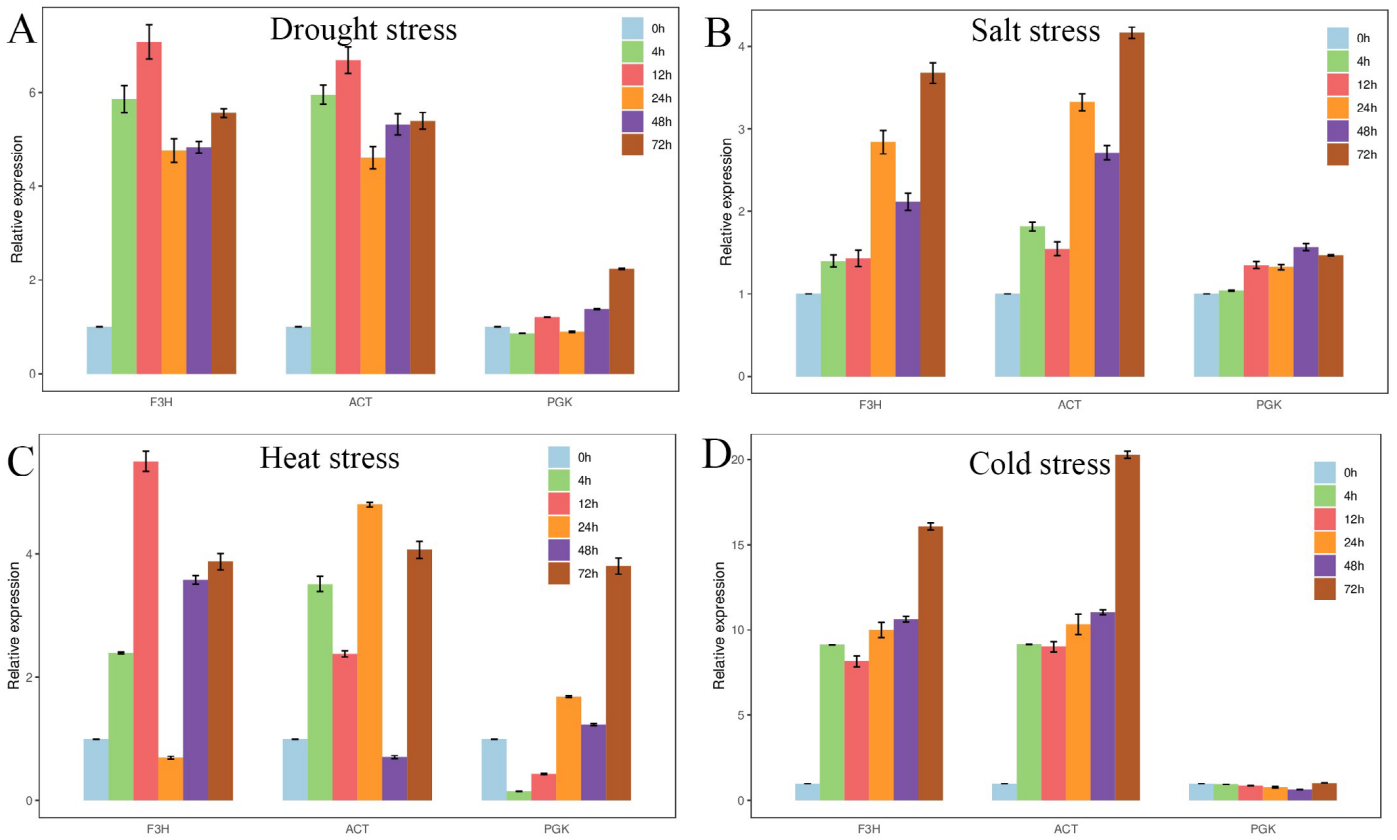
Expression of *IgP5CS* gene under different stresses using the selected reference genes for normalization. The most stable reference genes (*ACT* and *F3H*) and the most unstable gene (*PGK*) were selected as the normalization factors. Error bars show the standard error calculated from three biological replicates.

As shown in Figure 8, the expression of *IgP5CS* exhibited similar expression trends, when *ACT* and *F3H* were used for normalization. By comparison, the use of the unstable reference gene *PGK* led to a different expression pattern of *IgP5CS*. Therefore, unstable reference genes may lead to a deviation in the expression of the target gene and affect the accuracy of the experiment, which further verifies the reliability of using *ACT* and *F3H* as reference genes.

## 4 Discussion

qRT‐PCR is a highly accurate, simple, specific, and sensitive method for analyzing gene expression (Deng et al. 2016). The accuracy and stability of gene expression data mainly depend on the stability of reference genes (Xiao et al. 2014). In the case of plants, no reference gene was universally accepted under all test conditions or across various tissues (Hruz et al. 2011; Razavi et al. 2019). The expression levels of commonly used reference genes differs among different plant species, including *TUB*, *EF1α*, *ACT*, and *GADPH* (G. Wang et al. 2019; Zhang et al. 2022). However, there have been reports indicating that reference genes not only play a role in maintaining fundamental cellular functions but also actively contribute to various other cellular processes (De Vega‐Bartol et al. 2013). As a result, their expression levels exhibit significant variation across different experimental conditions. In *Schima superba*, *AP‐2* exhibited the highest stability for both cold and drought stresses, and *eIF‐5α* exhibited the highest stability for salt stress (Yao et al. 2022). In *Arabidopsis*, *Actin* exhibited higher stability under biotic and abiotic stress but showed relatively lower stability during developmental stages (Czechowski et al. 2005). Therefore, it is essential to validate the most suitable reference genes for specific conditions before utilizing them for normalization.

*I. germanica* L., a highly ornamental species of the genus *Iris*, exhibits a strong abiotic stress resistance, including drought, high temperatures, and low temperatures (Zhang et al. 2021). The current research on *I. germanica* L. primarily focused on the following aspects: physiological and biochemical measurement (Zhao et al. 2021), genetic diversity (Li et al. 2020), phenotypic analysis (Fan et al. 2022), and investigation of the flowering mechanism (Fan et al. 2020). Due to limited information on reference gene stability, few studies have been conducted on stress‐resistant mechanisms in *I. germanica* L. More and more researchers have reported the stability of gene expression across different plant species and under different stress conditions (Renganathan et al. 2023; Zhang et al. 2018; Zhang et al. 2017). However, to date, no comprehensive investigation of reference genes in *Iris* species across different abiotic stresses has been reported.

This research identified the suitable reference genes in *I. germanica* L. under various abiotic stresses. 9 reference genes were chosen from *I. germanica* L. to evaluate their stability in expression under drought, salinity, heat, and cold treatment. The ranking order calculated by different algorithms showed slight differences, due to the different statistical methods for assessing reference genes (Duan et al. 2017; Dudziak et al. 2020). NormFinder computes the SV, whereas GeNorm calculates the *M* value through pairwise variation, with both methods aiming to achieve minimal values that indicate optimal expression stability. BestKeeper evaluates the reference genes by analyzing SD and CV of Ct values across various samples (Pfaffl et al. 2004). The results obtained from GeNorm and NormFinder exhibited minimal variations, as both methods employ similar principles to evaluate reference genes. For example, *EF1α* was the most stable reference gene for salt stress according to GeNorm and NormFinder, whereas the best gene from BestKeeper was *ACT*. RefFinder allocates suitable weights to individual genes and computes their geometric mean to derive the conclusive ranking (He et al. 2019). We also used RefFinder to comprehensively evaluate the results from these three algorithms. The results from RefFinder exhibited a high degree of consistency with those obtained from NormFinder and GeNorm methods. Therefore, the most stable gene can serve as an internal reference gene for accurately correcting the expression levels of target genes under abiotic stress conditions.

To further demonstrate the reliability of the selected reference genes, we employed the two most stable genes (*ACT* and *F3H*) and one least stable gene (*PGK*) to standardize the expression levels of the *IgP5CS* gene. The expression of *IgP5CS* was significantly upregulated under drought, salt, and cold stress, which showed similar expression patterns of *PbP5CS*, *AtP5CS1*, and *SpP5CS* under abiotic stress conditions (Ma et al. 2022; Yang et al. 2021; Yoshiba et al. 1995). The expression level of *IgP5CS1* was not induced under heat stress, which is the same as the report that *AtP5CS1* was not responsive to high temperatures (Yoshiba et al. 1995). However, the use of an unstable reference gene (*PGK*) for normalization resulted in a significant deviation in the expression pattern of *IgP5CS*. The findings indicated that a more reliable gene can improve the precision of the results, and unstable reference genes can result in faulty qRT‐PCR results.

## 5 Conclusion

In this research, the expression stability of 9 candidate reference genes was evaluated by four different algorithms (GeNorm, Normfinder, BestKeeper, and RefFinder). Through the analysis of gene stability, *ACT* and *F3H* were identified as the most reliable reference genes under drought, heat, and cold stress. For salt stress, *EF1α* and *ACT* were the most stable reference genes. Furthermore, the expression patterns of *IgP5CS*‐validated *ACT* and *F3H* genes can serve as appropriate reference genes. Our research will significantly enhance greatly facilitate the identification of stress tolerance genes in *I. germanica* L. and other *Iris* plants.

## Author Contributions

**Yuan Yuan:** data curation (lead), formal analysis (lead), visualization (supporting), writing – original draft (lead). **Chungui Liu:** investigation (supporting), resources (supporting). **Jianzhong Bao:** data curation (supporting), formal analysis (supporting). **Fengtong Li:** conceptualization (equal), supervision (equal), visualization (equal).

## Conflicts of Interest

The authors declare no conflicts of interest.

## Data Availability

The data that support the findings of this study are available from the corresponding author upon reasonable request.
